# Influence of Alkaline Electrolyzed Water on the Strength, Shrinkage Behavior, and Microstructure of Alkali-Activated Fly Ash/Slag Composites

**DOI:** 10.3390/ma18245493

**Published:** 2025-12-06

**Authors:** Lili Li, Yaning Wu, Haozhe Wang, Zhen Zhu, Dingyuan Wu, Liang Wang, Ning Wang

**Affiliations:** 1 School of Civil Engineering and Architecture, Linyi University, Linyi 276000, China; lilili666819@163.com; 2 College of Civil Engineering & Architecture, Qingdao Agricultural University, Qingdao 266109, China; 3 College of Civil Engineering, Qingdao University of Technology, Qingdao 266033, China; 4 China Railway 16th Bureau Group Fourth Engineering Co., Ltd., Beijing 101400, China

**Keywords:** alkali activated, alkaline electrolyzed water, strength, microstructure, shrinkage behavior

## Abstract

In this study, the effects of highly active alkaline electrolyzed water (AEW) on the mechanical properties, shrinkage behavior, alkali activation reaction characteristics, and microstructure of alkali-activated fly ash/slag mortars at different alkali concentrations are systematically investigated, with ordinary tap water as the reference (OM group). The results showed that the EM group exhibited improved strength compared with the OM group. Specifically, the 28 d compressive and flexural strength of EM mortar at an alkali concentration of 4.0% were 13.5% and 7.5% higher than those of OM mortar, respectively. The 28 d drying shrinkage rate of the EM group was reduced by 7.3–11.2%. The EM group had a higher mass loss in the bounding water decomposition stage and a lower mass loss in the Ca(OH)_2_ and CaCO_3_ decomposition stages. XRD results showed that the EM group had a broader and stronger characteristic peak of N-A-S-H/C-A-S-H gel and a weaker characteristic peak of Ca(OH)_2_ than the OM group. The enhancement mechanism of AEW was attributed to its high ion activity, the dense microstructure formed by sufficient alkali activation reaction reduced the pore content, thereby improving the strength. The AEW-based alkali-activated material in this study can be widely used in green low-carbon infrastructure fields such as new energy infrastructure and ocean engineering.

## 1. Introduction

Against the backdrop of global “dual carbon” goals and resource recycling strategies, the construction industry is a key sector for energy consumption and carbon emissions. The production of traditional Portland cement faces issues such as high energy consumption, large carbon emissions, and severe environmental pollution [1], creating an urgent need for low-carbon, environmentally friendly, and high-performance new building materials. Alkali-activated geopolymer cementitious materials use industrial solid wastes (fly ash and blast-furnace slag) as main raw materials, realizing resource utilization of solid wastes through the action of alkali activators. Meanwhile, the geopolymers composed solely of precursors, without the addition of sodium silicate, exhibit carbon footprints significantly lower than Portland cement-based mixtures [2], making them ideal green cementitious materials to replace traditional cement. However, current alkali-activated solid waste-based mortars still face technical bottlenecks, including slow early strength development, excessively long setting time, poor interface bonding performance, and susceptibility to shrinkage and cracking [3,4,5], which limit their large-scale engineering application. Among these factors, the activity and characteristics of mixing water have a significant effect on the alkali activation reaction process, alkali activation reaction product formation, and the final performance of mortars. Traditional mixing tap water has low activity and cannot effectively accelerate the dissolution and reaction of solid waste particles [6]. Improving the performance of alkali-activated concrete through high-activity and environmentally friendly mixing water has become one of the key directions to break through this technical bottleneck.

At present, many researchers have studied the performance optimization of alkali-activated solid waste-based cementitious materials [7]. In terms of optimizing the activator system, research has mostly focused on the ratio control of composite alkali activators and cementitious material systems. H. Ma [8] used a mechanical activation method to improve the activity of fly ash and slag, promoting their reaction efficiency with activators and improving the strength of alkali-activated concrete. Z. Jiao [9] found that under low alkali concentration, higher fly ash content can significantly enhance pore size control, refine mesopores, and compact macroscopic structures, but it can lead to significant changes in structural stability. L. Araújo [10] indicated that high-temperature curing can increase the compressive strength of alkali-activated concrete by nearly 60% after 28 days, and high-energy input mixing methods can also improve compressive strength. H. Ma [11] found that alkaline-activated AAFS concrete samples exhibit excellent conductivity, the water distribution is the most important factor affecting the conductivity of AAFS samples, and the conductivity of AAFS can be optimized by adjusting pore connectivity and ion migration pathways. Y. Liu [12] found that with the increase in desert sand content, the reaction heat release rate of alkali-activated paste decreases, while the diffraction peak intensity of quartz, drying shrinkage, and quality loss of paste increase. Z. Zhu [13] believed that when the pH value of the alkaline-activated paste pore solution is high, it is easy for the material to fully react, resulting in dense and abundant reaction products in the interface transition zone. S. Guo [14,15] used Na_2_SO_4_ + Ca(OH)_2_ as an activator to study the effect of Ca(OH)_2_ on the early performance of alkali-activated slag concrete. They found that the addition of Ca(OH)_2_ increased the setting rate of alkali slag concrete and caused rapid slump loss, and the reaction rate during the reaction acceleration period of alkali slag concrete significantly increased. H Abdel-Gawwad [16,17] found that adding limestone powder to alkali-activated artificial slag mortar can reduce the yield stress and shear thinning degree of the mortar while increasing the rheological index of the mortar and improving its workability. J. Zhang [18] indicated that fly ash/slag content and w/b ratio are the main parameters affecting the long-term migration of chloride ions in AAFS concrete. In terms of mixing water modification, some studies have explored the application effects of special water, such as magnetized water and nano-modified water, confirming that they can optimize the workability and mechanical properties of mortar by changing the water molecule structure and enhancing activity [19,20,21,22]. The study of using highly active alkaline electrolyzed water as mixing water to enhance alkali-activated polymer materials is still in its infancy. Alkaline electrolyzed water, due to its special electrochemical properties such as high pH value, high activity hydroxide ions, and small molecular cluster structure, can enhance the dissolution and reaction kinetics on the surface of particles, promote the hydration process, and improve the workability of paste materials [23,24,25]. Meanwhile, the preparation process of highly active alkaline electrolyzed water is clean, with low energy consumption, and does not require the addition of strong alkali, which can avoid the corrosion problem when traditional mixing water is used in combination with activators [26,27]. It helps to reduce the production cost of alkali-activated geopolymer mortar, improve its environmental friendliness, and provide a new technological path for the promotion and application of industrial solid waste-based low-carbon building materials. However, the relationship between the activity of alkaline electrolyzed water and the macroscopic properties and microstructure of alkali-activated fly ash/slag mortar is not yet clear.

In summary, in this study, a method for preparing alkali-activated fly ash mineral powder mortar based on highly active alkaline electrolyzed water (AEW) as mixing water is developed. By optimizing the preparation process parameters of highly active AEW, the influence of highly active AEW as mixing water on the workability, mechanical properties, and durability of alkali-activated fly ash/slag material was systematically studied. The alkali activation reaction product composition, microstructure characteristics, and interface bonding state were analyzed by testing methods such as X-ray diffraction, SEM scanning electron microscopy, and thermogravimetric analysis, revealing the micro mechanism of AEW strengthening the alkali activated reaction of fly ash/slag, having important practical significance for promoting the promotion and application of alkali activated solid waste based green cementitious materials.

## 2. Experimental Program

### 2.1. Experimental Materials

#### 2.1.1. Mineral Admixtures

In this study, the S95 blast furnace slag (provided by Qingdao Qingjian Group Co., Ltd., Qingdao, China) was used as the precursor for alkali-activated material, with a specific surface area of 425 m^2^/kg and a density of 2.87 g/cm^3^; II-class fly ash (produced by Weifang Huadian Co., Ltd., Weifang, China) was used, with a density of 2.25 g/cm^3^, a water demand ratio of 0.96%, a loss on ignition of 0.9%, and a specific surface area of 3950 cm^2^/g. The XRF chemical composition analysis results of slag and fly ash are shown in Table 1.

#### 2.1.2. Alkaline Activator

The liquid water glass provided by Qingdao Ruihengda Chemical Materials Co., Ltd. (Qingdao, China) was used as the alkali activator; the water glass modulus was 3.4, the Baume degree was 39.4, and the liquid content was 36.41%. According to the mix proportion ratio requirements of the experiment, the water glass modulus can be adjusted by adding NaOH. The analytical purity of the NaOH used was provided by Dezhou Runxin Experimental Instrument Co., Ltd. (Dezhou, China), with a purity of ≥96.0%.

#### 2.1.3. Alkaline Electrolyzed Water (AEW)

**Figure 1:**
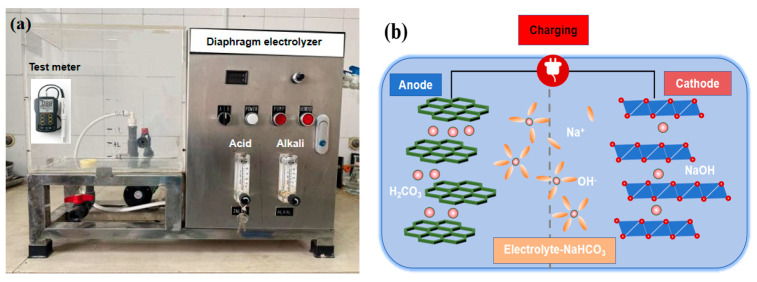
Electrolyzed water equipment and its schematic diagram (**a**): Diaphragm electrolyzer; (**b**): schematic diagram.

In this study, NaHCO_3_ was used as the electrolyte with a concentration of 0.05%, and direct current was applied to the diaphragm electrolysis cell (provided by Shanghai Anyutai Environmental Protection Technology Co., Ltd., Shanghai, China) for electrolysis. During the electrolysis process, oxidation and reduction reactions occurred at the anode and cathode of the diaphragm electrolysis cell, respectively [28]. Alkaline electrolyzed activated water was generated at the cathode, with the main component being hydroxide ions (OH^−^); acidic electrolyzed activated water was generated at the anode, with the main components being hydrogen ions (H^+^) and carbonic acid. A water quality tester was used to measure the pH value, oxidation–reduction potential (ORP), and total dissolved solids (TDS) of the water. The specific parameters of different waters are shown in Table 2. The pH value mainly reflects the acidity or alkalinity of the water, the ORP value reflects the redox capacity of the water [29], and the TDS mainly reflects the conductivity and hardness of the water. The higher the TDS, the higher the conductivity and hardness of the water. As can be seen from Table 2, NaOH is mainly generated on the cathode side of the electrolytic cell. The alkaline electrolyzed water has a pH value of 10.0 and a TDS value as high as 106, indicating high reaction activity. Therefore, in this study, alkaline electrolyzed water is used to improve the performance of alkali-activated fly ash/slag materials. The electrolyzed water equipment and its schematic diagram are shown in Figure 1a,b.

#### 2.1.4. Fine Aggregate

The machine-made sand prepared by Qingdao Qingjian Group Co., Ltd. (Qingdao, China) was used as fine aggregate; its apparent density was 2648 kg/m^3^, with a fineness modulus of 2.62, a mud content of 0.9%, a crushing index of 10.9%, and a water absorption rate of 1.7%, which meets the standard requirement for medium sand.

#### 2.1.5. Water-Reducing Admixture

The water-reducing admixture used was the high-performance polycarboxylate superplasticizer provided by Jiangsu Subot New Material Co., Ltd. (Nanjing, China), with a water reduction rate of 25% and a content of 1.5% of the alkali-activated material.

### 2.2. Mix Proportion Design of Alkaline-Activated Material

In this study, the ordinary tap water (OTW) was used as the blank control group (OM), alkaline electrolyzed water series was used as the experimental group (EM), and a composite system of slag and fly ash was used as the alkali-activated precursor. The ratio of slag to fly ash was designed to be 1:4, with two binder material/sand ratio series (c/s ratio 1:2 and 1:3), respectively. Two alkali-activated slag/fly ash material systems were designed, with a total binder material amount of 600 kg/m^3^ and a sand amount of 1200 kg/m^3^ for series 1. The total amount of binder material was 500 kg/m^3^, with a sand content of 1500 kg/m^3^ used in series 2. On this basis, alkali concentration gradients of 3.0%, 3.5%, and 4.0% were set, achieving precise alkalinity control through the compounding of water glass (1.0 modulus, Na_2_O content of 13.5%) and NaOH solution (3 mol/L concentration). Meanwhile, the sol ratio was fixed at 0.6 to ensure that the total liquid mass of the electrolyzed water group (EM) and the tap water group (OM) was consistent, thereby eliminating the interference of mixing water quantity differences on the experimental results. Through this standardized proportion design, a total of 12 comparative experimental schemes were ultimately constructed. The detailed mix proportion ratio design is shown in Table 3.

### 2.3. Experimental Methods

**Figure 2:**
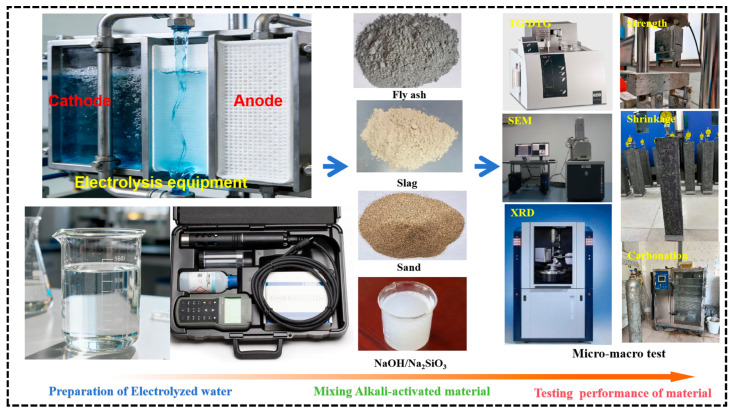
The detailed experimental procedure of this study.

The cubic specimens with dimensions of 150 mm × 150 mm × 150 mm and prismatic specimens with dimensions of 100 mm × 100 mm × 400 mm were prepared. Each group consists of 3 test specimens. The tests for the compressive strength and flexural strength of alkali-activated specimens were conducted within the specified curing age period. The shrinkage test used a test specimen with dimensions of 100 mm × 100 mm × 515 mm, and test probes were pre-embedded at both ends of the formwork. After molding, the test specimen was moved into a curing room, and immediately after 3 days, it was transferred to a constant temperature and humidity curing environment with a temperature of (20 ± 2) °C and a relative humidity of (60 ± 5)%. The initial length of the test block was measured. After curing to the specified test age, the shrinkage values were measured at 1 d, 3 d, 7 d, 14 d, 28 d, 45 d, 60 d, and 90 d in sequence. In the carbonation test, the dimensions of the prismatic specimens were 100 mm × 100 mm × 400 mm, the concentration of CO_2_ in the carbonation test chamber was controlled at 18~22%, the humidity at 65~75%, and the temperature at 15~25 °C. Carbonation depth measurements were carried out within the specified curing age period. In terms of microscopic experiments, the alkali-activated samples were cured for 28 days and then removed and crushed into small particle samples ranging from 2.5 mm to 5.0 mm. The samples were soaked in acetone for 24 h, dried in a drying oven for 12 h, and then placed in a vacuum chamber to ensure complete cessation of the alkali activation reaction in the particle samples. After complete drying, the particle samples were ground into powder and sieved through a 40 μm sieve as the test sample. The mineral composition peaks of the alkali activation reaction products were analyzed using a thermal gravimetric analysis (TG/DTG), the mineral composition of the paste reaction products was analyzed using X-ray diffraction (XRD), and the microstructure and pore distribution of different pastes were observed using scanning electron microscopy (SEM) analyses. The experimental procedure is shown in Figure 2.

## 3. Results and Discussion

### 3.1. Workability of AEW-Based Alkali-Activated Composites

**Figure 3:**
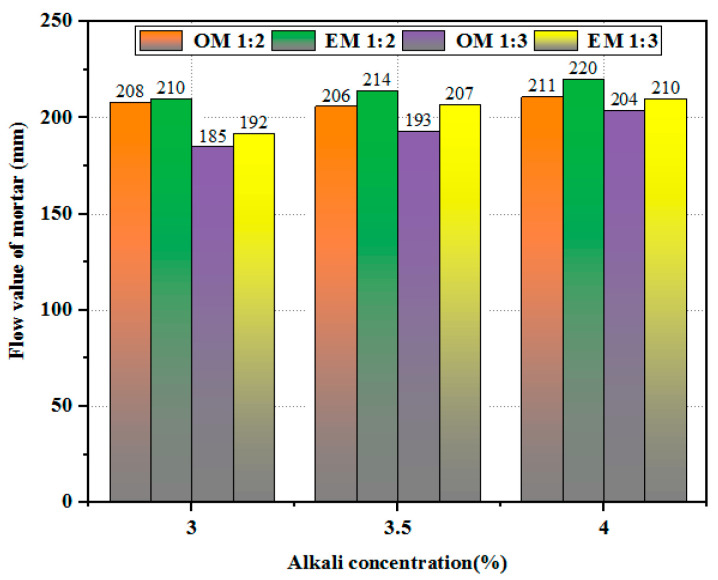
Fluidity of different series of alkali-activated materials at different alkali concentrations.

Figure 3 shows the workability of AEW-based alkali-activated composites under different alkali concentrations. It can be seen that for the OM series, the fluidity of the mortar with a c/s ratio of 1:3 is more sensitive to alkali concentration, while the fluidity of the mortar with a c/s ratio of 1:2 remains almost unchanged, indicating that the workability of OM mortar is less responsive to changes in alkali concentration. For the EM series, regardless of the c/s ratio being 1:2 or 1:3, an increase in alkali concentration can enhance fluidity, and the increase is more significant when the c/s ratio is 1:2, indicating that the workability of EM alkali-activated mortar is overall more efficient than increases in alkali concentration, and the positive impact of alkali concentration is more prominent in the case of a lower c/s ratio. Meanwhile, as the c/s ratio increases, the amount of alkali-activated material in the paste increases, which can fully encapsulate the fine aggregates, thereby improving the workability of the mortar. When the alkali concentration is 4%, as the c/s ratio increases from 1:3 to 1:2, the fluidity of the OM series increases from 204 mm to 211 mm, while the fluidity of the EM series increases from 210 mm to 220 mm. Overall, when the alkali concentration increases, the improvement in workability of the EM mortar is more universal, and its fluidity is significantly better than that of the OM series control group.

### 3.2. Flexural Strength of AEW-Based Alkali-Activated Composites

**Figure 4:**
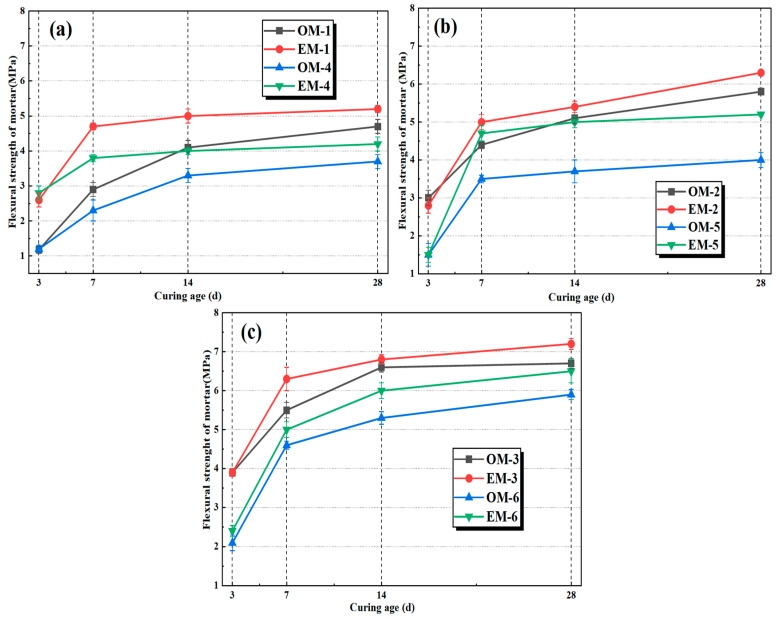
Flexural strengths of alkali-activated mortar under different alkali concentrations: (**a**): alkali concentration of 3.0%; (**b**): alkali concentration of 3.5%; (**c**): alkali concentration of 4.0%.

Figure 4 shows the flexural strength development of different alkali-activated composites under different alkali concentrations. As shown in Figure 4a–c, the flexural strengths of both series of alkali-activated mortars increase with the curing age. Under the conditions of alkali concentrations of 3.0%, 3.5%, and 4.0%, the flexural strength of EM mortar is significantly higher than that of OM mortar at all curing ages. Meanwhile, with the increase in alkali concentration, the flexural strength of both OM and EM mortars increases continuously. When the alkali concentration is 3.0% and the c/s ratio is 1:2, the 28-day flexural strengths of EM-1 and OM-1 reach 5.2 MPa and 4.0 MPa, respectively; while the alkali concentration is 4.0%, the 28-day flexural strengths of EM-3 and OM-3 reach 7.0 MPa and 6.6 MPa, respectively. When the alkali concentration is low, there is a significant difference in early strength between OM and EM mortar at the same c/s ratio. When the alkali concentration reaches 4.0%, the difference in early strength weakens.

This is mainly because the OH^−^ concentration provided by the alkali activator is the core driving force of the alkali activation reaction. A significant increase in OH^−^ concentration can promote the dissolution of more active SiO_2_ and Al_2_O_3_, making the activation more sufficient and generating more N-A-S-H/C-A-S-H gels [30]. These gels fill the gaps between fine aggregates and alkali-activated binder materials, forming a more continuous network structure, which improves the overall compactness and strength of the mortar. In addition, regardless of the c/s ratio, the strength development of EM mortar is more stable, and the peak value is higher, indicating that AEW, as an activation medium, can more effectively improve the flexural strength of alkali-activated mortar, and this advantage is also consistent under different alkali concentrations.

### 3.3. Compressive Strength of AEW-Based Alkali-Activated Composites

**Figure 5:**
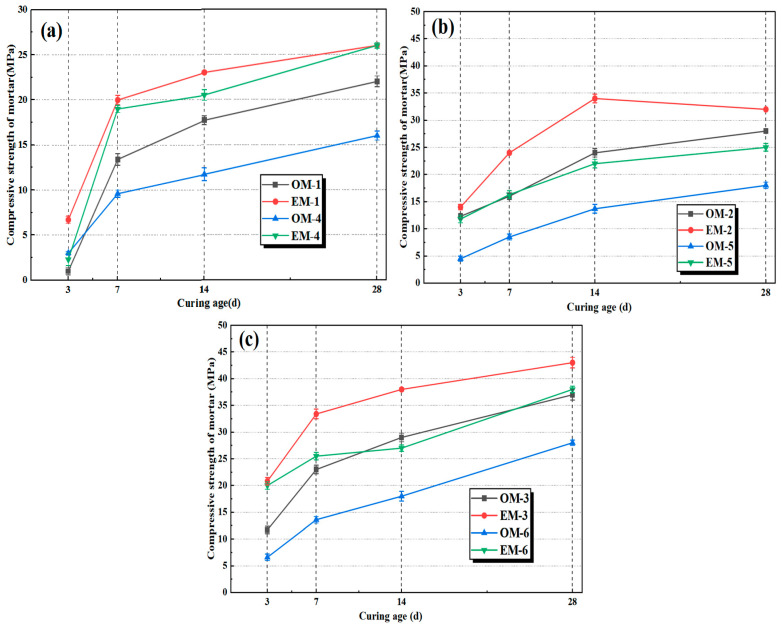
Compressive strengths of alkali-activated mortar under different alkali concentrations: (**a**): alkali concentration of 3.0%; (**b**): alkali concentration of 3.5%; (**c**): alkali concentration of 4.0%.

Figure 5 shows the compressive strengths of different alkali-activated composites under different alkali concentrations. It can be indicated that, similar to the development results of flexural strength of mortar, under the conditions of different alkali concentrations of 3.0%, 3.5%, and 4.0%, the compressive strength of EM mortar in the AEW series is significantly higher than that of OM mortar at all curing ages. When the alkali concentration is 3.0%, the 28-day strength peak of EM-1 reaches 26 MPa, while that of OM-1 reaches 22 MPa; the strength growth rate of EM-1 is around 18%. As the alkali concentration increases, the strength gap between EM and OM further widens, and the strength development of EM mortar is more stable with a higher peak value. When the alkali concentration is 4.0%, the strength peak of EM-3 (42 MPa) is significantly higher than that of OM-3 (37 MPa), the growth rate is 13.5%, and an adequate stimulation of the reaction causes the products to fill the pores more tightly, thereby improving strength.

### 3.4. Shrinkage of AEW-Based Alkali-Activated Composites

**Figure 6:**
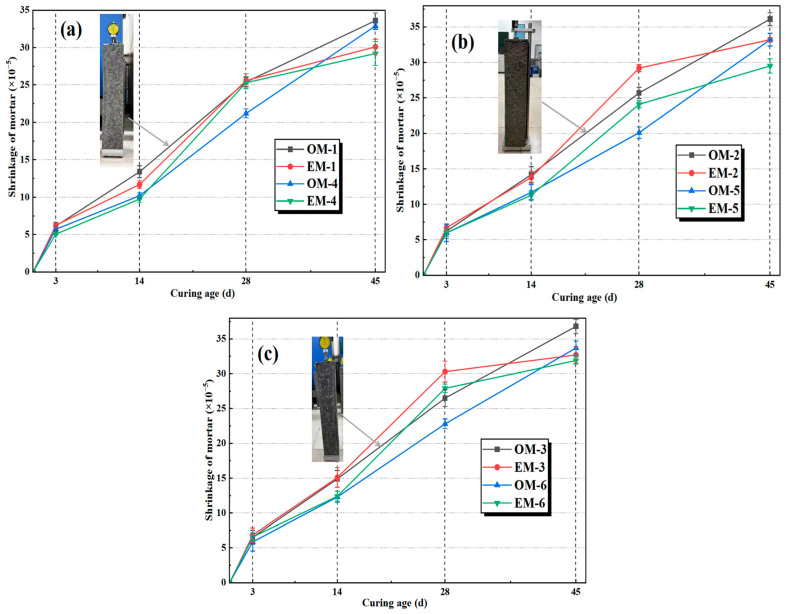
Shrinkage of alkali-activated mortar under different alkali concentrations: (**a**): alkali concentration of 3.0%; (**b**): alkali concentration of 3.5%; (**c**): alkali concentration of 4.0%.

Figure 6 shows the shrinkage of different alkali-activated composites under different alkali concentrations. Under different alkali concentration conditions, the shrinkage rate of EM mortar is lower than that of OM mortar at all curing ages. The main reason lies in the highly active activation of AEW, which can further promote the early formation of products. A large amount of generated gel tightly fills the pores, promoting the early densification of the microstructure. This not only reduces the channels for water migration but also enhances the constraint effect on shrinkage through the dense skeleton, fundamentally suppressing the development of late-stage shrinkage [31]. In contrast, due to the delayed activation reaction of OM mortar, the driving force for late-stage shrinkage persists, resulting in a higher shrinkage rate. Taking the c/s ratio of 1:2 as an example, compared with the control group, the 45 d shrinkage rate of EM-1 decreased by about 11.1%, and the shrinkage rate of EM-3 decreased by about 9.3%.

### 3.5. Carbonation of AEW-Based Alkali-Activated Composites

**Figure 7:**
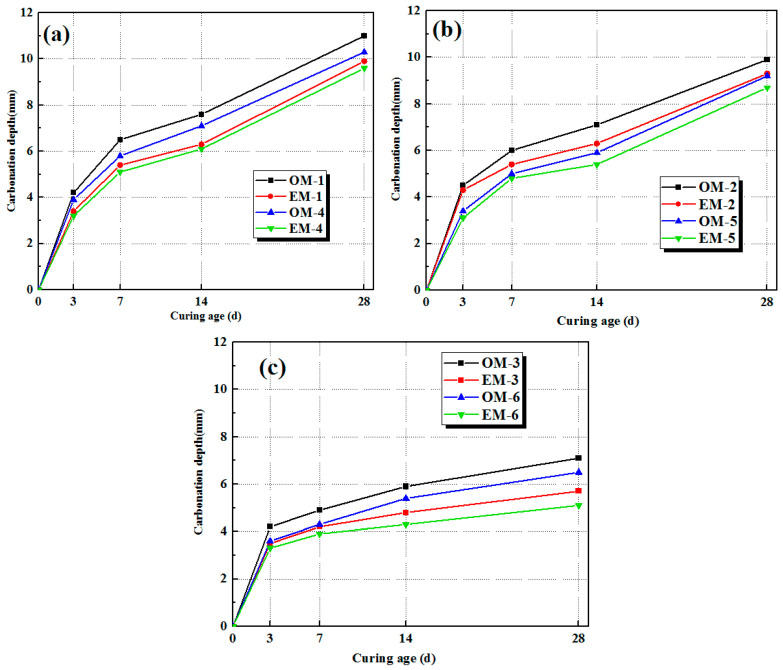
Carbonation depths of alkali-activated mortar under different alkali concentrations: (**a**): alkali concentration of 3.0%; (**b**): alkali concentration of 3.5%; (**c**): alkali concentration of 4.0%.

Figure 7 shows the carbonation depth curves of the alkali-activated materials under different alkali concentrations with curing ages. It can be indicated that the degree of carbonation reaction decreases with the increase in the c/s ratio, and the carbonation depth of each EM group is smaller than that of the OM group. It can be seen from Figure 7a that when the alkali concentration is 3.0%, the maximum 28-day carbonation depth of the OM-4 group reaches 10.9 mm, and that of the EM-4 group reaches 9.8 mm. With the increase in the c/s ratio, the carbonation depth decreases, with the OM-1 group reaching 10.2 mm and the EM-1 group reaching 9.7 mm. By comparing with Figure 7c, as the alkali concentration increases to 4.0%, the carbonation rates of both the OM and EM groups slow down significantly, and the carbonation depth decreases noticeably. The maximum 28-day carbonation depth of the OM-6 group is only 7.1 mm, and that of the EM-6 group is only 5.8 mm. Therefore, the carbonation depth of alkali-activated mortar decreases with the increase in alkali concentration. AEW can further reduce the carbonation depth of concrete and slow down the carbonation rate of the alkali-activated paste. This is mainly because AEW improves the reaction degree of alkali-activated materials, making the paste structure more compact, thereby reducing the intrusion degree of CO_2_. At the same time, the alkaline environment of AEW increases the alkali reserve in the concrete structure, maintaining the stability of the alkaline environment during carbonation, which is beneficial to the improvement of the carbonation resistance of mortar.

### 3.6. Microscopic Properties of AEW-Based Alkali-Activated Composites

#### 3.6.1. Alkali Activation Reaction Characteristics

**Figure 8:**
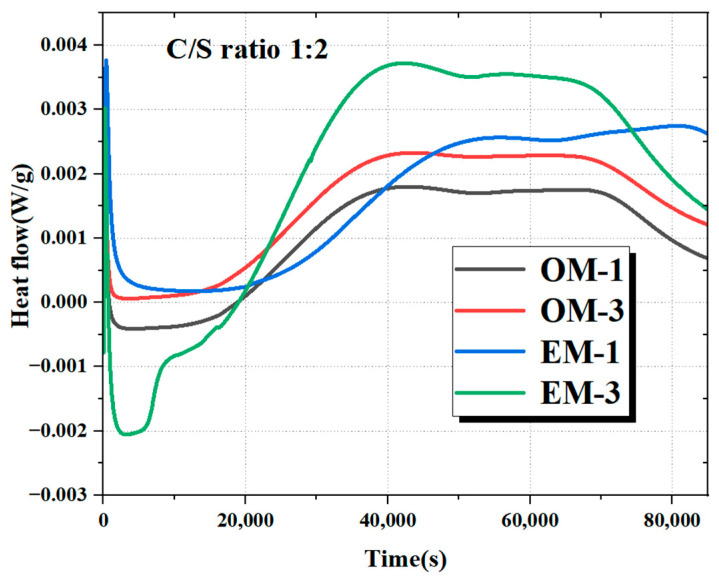
Reaction heat curves of different modified RWF-SAC materials.

When the c/s ratio is 1:2 and the alkali concentrations are 3.0% and 4.0%, respectively, the reaction exothermic curves of OM and EM mortar under different alkali concentrations are shown in Figure 8. It can be seen that the alkali activation reaction heat flow curves of the OM mortar are both generally stable, while the peak value of the flow curve of the EM mortar fluctuates greatly. The instantaneous early reaction rate and the highest heat flow peak value are significantly higher than those of the OM mortars, and the EM-3 group has the highest reaction heat flow peak value. This may be due to the high activity and small molecule adsorption of AEW, which can enhance the reactivity of mineral powder and fly ash particles with water, accelerate the dissolution and condensation process of the glass phase, promote the rapid growth of products such as N-A-S-H and C-A-S-H in alkaline-activated materials, and release additional reaction heat [32]. Meanwhile, the higher the alkali concentration, the greater the activity of the alkali-activated reaction, accelerating the initial reaction rate and resulting in the highest peak heat flow of the EM-3 mortar.

#### 3.6.2. Thermogravimetric Analysis (TG/DTG)

**Figure 9:**
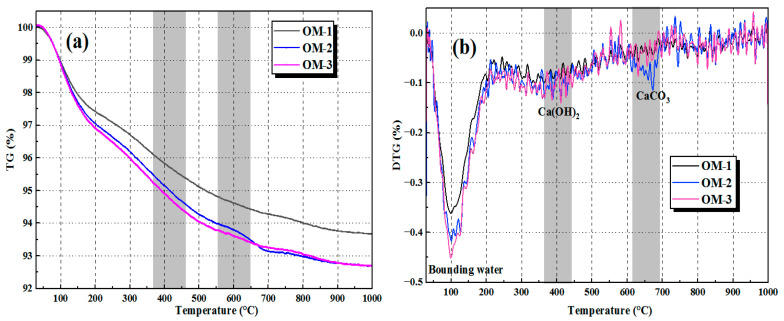
TG/DTG curves of OM alkali-activated materials: (**a**) TG curve; (**b**) DTG curve.

**Figure 10:**
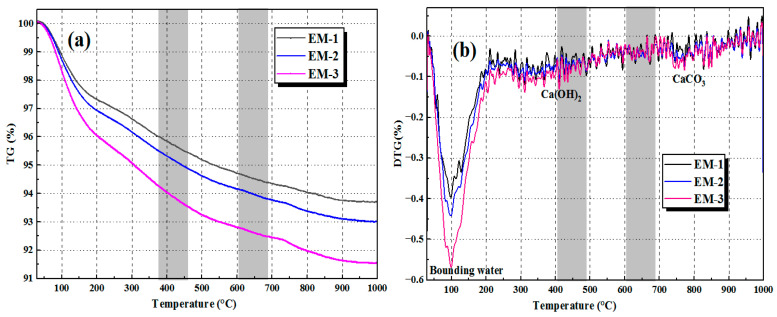
TG/DTG curves of EM alkali-activated materials: (**a**) TG curve; (**b**) DTG curve.

Figure 9 and Figure 10 show the TG-DSC curves of OM and EM mortars at the c/s ratio of 1:2, respectively. It can be seen from the comparison of the figures that the bound water comes from the chemically bound water of reaction products (such as N-A-S-H/C-A-S-H gels), and its content is related to the degree of alkali activation reaction. Comparing the bound water peak in the DTG curves of Figure 9b and Figure 10b, the peak intensity and range of the EM group are more significant, indicating that AEW in EM mortar promotes the alkali-activated reaction of fly ash and slag, making the dissolution and polycondensation reaction more sufficient and generating more reaction products. At the same time, the peak intensity of Ca(OH)_2_ in the EM mortar is significantly lower than that in the OM group, indicating that more Ca(OH)_2_ in the EM group participates in the pozzolanic reaction, generating more N-A-S-H gels and improving the compactness and strength of the mortar [33]. The peak intensity of CaCO_3_ in the EM group is also lower than that in the OM group, indicating that the EM group mortar has higher compactness, making it difficult for CO_2_ to invade and having stronger carbonation resistance. Moreover, as the alkaline concentration increases, the bound water and reaction products in the OM and EM groups also increase. Therefore, the EM mortar has a higher mass loss in the bound water stage (100~300 °C), while the mass loss is lower in the reaction product decomposition stage (after 300 °C), and the generated gel products are more stable, which confirms that the EM group has a more sufficient alkali activation reaction and more stable reaction products.

#### 3.6.3. XRD Analysis

**Figure 11:**
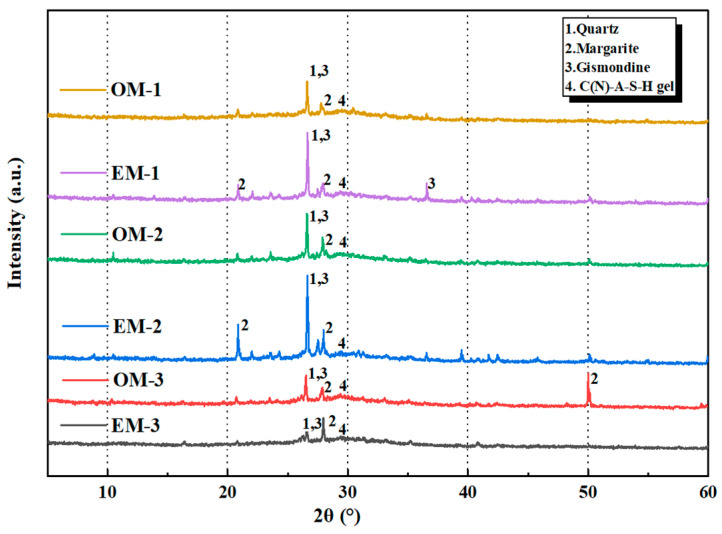
XRD patterns of different alkali-activated materials.

Figure 11 shows the XRD patterns of the reaction products of OM and EM mortar at the c/s ratio of 1:2. From Figure 11, it can be seen that crystalline mineral phases such as Margarite and Gismondine are both present in the alkali-activated material pastes of OM and EM groups. This is mainly formed by the depolymerization and re-polymerization reactions of silicon aluminum calcium components in slag and fly ash in alkaline environments. At high alkaline concentrations, ions in the solution tend to form ordered crystal structures. Under the same alkaline concentration conditions, the intensity of characteristic peaks of paste in the EM group is generally higher than that in the OM group, indicating that alkaline electrolyzed water can provide a more alkaline environment and higher reaction activity, promote the generation of characteristic products in alkali-activated reactions, and contribute to the performance enhancement of EM group materials in the later period. However, the peak intensity difference between the EM-3 and OM-3 groups is relatively reduced, which may be due to the fact that the high alkaline concentration of 4.0% itself provides a strong driving force for alkali-activated reactions of the material, which, to some extent, weakens the gain effect of alkaline electrolyzed water. In addition, crystalline mineral phases quartz (SiO_2_) are found to be difficult to completely activate using alkali material, showing characteristic sharp peaks [34]. The presence of these peaks indicates that some crystalline minerals in the raw materials do not participate in the reaction.

#### 3.6.4. SEM Microscopic Morphology

**Figure 12:**
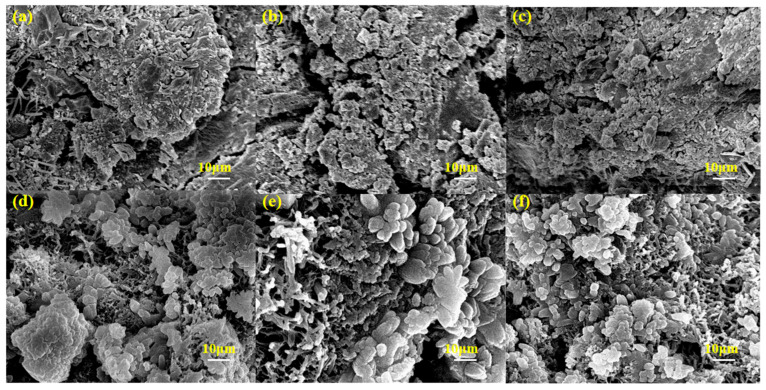
SEM images of different alkali-activated fly ash/slag materials: (**a**) OM-1; (**b**) OM-2; (**c**) OM-3; (**d**) EM-1; (**e**) EM-2; (**f**) EM-3.

Figure 12 observes the SEM images of OM mortar and EM mortar at the c/s ratio of 1:2, respectively. From Figure 12a–c, the cementitious products of mortar in the OM group are mostly flocculent and lamellar stacked, with relatively single morphology, obvious cracks, general structural compactness, and certain voids in the accumulation of products. With the increase in alkali concentration, cracks still exist in the paste, the agglomeration morphology of products is relatively rough, and the compactness is not significantly improved. There are still loose areas in the structure, and the overall compactness is not significantly improved. In contrast, from Figure 12a–c, the reaction products of the EM group are mostly granular and clustered, with more regular morphology, no obvious large cracks, a tighter structure, and significantly fewer voids in product accumulation than those in the OM group with the same concentration [35]. Moreover, as the alkali concentration increases from 3.0% to 4.0%, the cohesiveness between particles is enhanced, the compactness is further improved, and a more continuous spatial network structure is formed.

It can be seen that alkaline electrolyzed water (AEW), as an excitation medium, can significantly improve the microstructural compactness of alkali-activated fly ash/slag mortar and optimize the morphology of products under the same condition of alkali concentration; and in the alkaline electrolyzed water system, the increase in alkali concentration has a more obvious optimization effect on the structure and alkali activation reaction products.

## 4. Conclusions

In this study, using ordinary tap water series as a blank control, the effects of AEW on the mechanical properties, durability, and micro-performance of alkali-activated fly ash/slag composites are investigated at different c/s ratios and alkali concentrations, to improve the performance of alkali-activated fly ash/slag composites. The conclusions obtained are drawn as follows:

(1) Highly active alkaline electrolyzed water can enhance the mechanical properties of alkali-activated fly ash/slag mortar (AAFSM). At the same alkali concentration, the EM group outperforms the OM group in both compressive and flexural strengths. The EM mortar at an alkali concentration of 4.0% achieves the optimal performance, and 28 d compressive and flexural strength increased by 13.5% and 7.5%, respectively. The 28 d drying shrinkage rate of the EM group is reduced by 7.4–11.2% more than the OM group.
(2) AEW promotes the alkali activation reaction process and optimizes the microstructure of AAFSM. TG/DTG results show the EM group has higher bound water mass loss and lower Ca(OH)_2_/CaCO_3_ mass loss. XRD analysis confirms the EM group has stronger N-A-S-H/C-A-S-H gel peaks and weaker peaks of unreacted minerals, verifying a more adequate alkali activation reaction.
(3) A critical finding is that 4.0% alkali concentration maximizes AEW’s activation efficacy, balancing strength improvement and shrinkage inhibition. This concentration maximizes the acceleration of silicon–aluminum component dissolution in fly ash and slag by AEW’s high ion activity, promotes the formation of high-quality N-A-S-H/C-A-S-H gels, and constructs a dense microstructure, thereby comprehensively improving the strength, shrinkage resistance, and durability of AAFSM.
(4) This study validated AEW as a high-efficiency, eco-friendly activator for alkali-activated materials (AAMs), addressing the need for performance enhancement of solid waste-based AAMs and advancing low-carbon construction materials. However, this study focuses on mortar under standard conditions; future research will further optimize AEW preparation parameters to extend AEW’s application to alkali-activated concrete with other industrial solid wastes to broaden its sustainability value.

## Figures and Tables

**Table 1:** The XRF chemical composition analysis results of slag and fly ash (%).

Chemical Composition	CaO	Al_2_O_3_	SO_3_	SiO_2_	MgO	Fe_2_O_3_	TiO_2_	Na_2_O	Others
Fly ash	2.56	29.71	0.37	56.49	1.48	4.33	1.75	0.48	2.83
Blast furnace slag	38.32	12.28	1.14	32.07	7.64	0.47	1.63	0.25	6.20

**Table 2:** The performance parameters of different types of water.

Performance Parameters	Ordinary Tap Water	Acid Electrolyzed Water	Alkaline Electrolyzed Water
pH value	7.8	6.7	10.0
ORP value (oxidation–reduction potential)	317	353	−226
TDS (mg/L)	69	30	107

**Table 3:** Mix proportions of alkaline-activated materials.

Cementitious- Sand Ratio	Alkali Concentration (%)	Mark	Slag (kg/m^3^)	Fly Ash (kg/m^3^)	Sand (kg/m^3^)	NaOH (kg/m^3^)	Water Glass (kg/m^3^)	Water (kg/m^3^)
1:2	3.0	OM-1	480	120	1200	16.1	64.4	279.5
	3.5	OM-2	480	120	1200	18.8	75.2	266
	4.0	OM-3	480	120	1200	21.5	85.9	252.6
1:3	3.0	OM-4	400	100	1500	13.4	53.7	232.9
	3.5	OM-5	400	100	1500	15.7	62.6	221.7
	4.0	OM-6	400	100	1500	17.9	71.6	210.5
1:2	3.0	EM-1	480	120	1200	16.1	64.4	279.5
	3.5	EM-2	480	120	1200	18.8	75.2	266
	4.0	EM-3	480	120	1200	21.5	85.9	252.6
1:3	3.0	EM-4	400	100	1500	13.4	53.7	232.9
	3.5	EM-5	400	100	1500	15.7	62.6	221.7
	4.0	EM-6	400	100	1500	17.9	71.6	210.5

## Data Availability

The original contributions presented in this study are included in the article. Further inquiries can be directed to the corresponding authors.
